# SSA-mediated selection marker gene activation enhances relative gene targeting efficiency in plants

**DOI:** 10.1093/hr/uhaf196

**Published:** 2025-11-05

**Authors:** Dali Kong, Yiqiu Cheng, Yongping Ke, Xiaofei Dang, Xin Liu, Congnawei Wang, Chaofeng Huang, Ruiqiang Ye, Daisuke Miki

**Affiliations:** CAS Center for Excellence in Molecular Plant Sciences, Chinese Academy of Sciences, Shanghai 200032, China; University of Chinese Academy of Sciences, Beijing 100049, China; Guangdong Key Laboratory for Genome Stability and Disease Prevention, Shenzhen University Medical School, Shenzhen, Guangdong 518060, China; CAS Center for Excellence in Molecular Plant Sciences, Chinese Academy of Sciences, Shanghai 200032, China; University of Chinese Academy of Sciences, Beijing 100049, China; CAS Center for Excellence in Molecular Plant Sciences, Chinese Academy of Sciences, Shanghai 200032, China; University of Chinese Academy of Sciences, Beijing 100049, China; CAS Center for Excellence in Molecular Plant Sciences, Chinese Academy of Sciences, Shanghai 200032, China; CAS Center for Excellence in Molecular Plant Sciences, Chinese Academy of Sciences, Shanghai 200032, China; University of Chinese Academy of Sciences, Beijing 100049, China; CAS Center for Excellence in Molecular Plant Sciences, Chinese Academy of Sciences, Shanghai 200032, China; University of Chinese Academy of Sciences, Beijing 100049, China; CAS Center for Excellence in Molecular Plant Sciences, Chinese Academy of Sciences, Shanghai 200032, China; CAS Center for Excellence in Molecular Plant Sciences, Chinese Academy of Sciences, Shanghai 200032, China; CAS Center for Excellence in Molecular Plant Sciences, Chinese Academy of Sciences, Shanghai 200032, China; SUAT Institute of Emerging Agricultural Technology, Shenzhen University of Advanced Technology, Shenzhen, Guangdong 518107, China; Kihara Institute for Biological Research, Yokohama City University, Yokohama, Kanagawa 244-0813, Japan

## Abstract

The precise manipulation of genome sequences through gene targeting (GT) is beneficial; however, the low efficiency of homology-directed repair (HDR) in seed plants has made GT difficult to achieve. Generation of double-strand breaks (DSBs) at the target DNA site of interest represents a promising approach to facilitate HDR-mediated GT in organisms. Despite recent advances, GT remains a significant challenge in seed plants. To address these challenges, we propose that the efficiency of CRISPR/Cas9-mediated GT could be enhanced by the exclusive selection of plants that exhibit high levels of HDR activity. To test this hypothesis, a surrogate screening system was developed, which consists of a nonfunctional split-selection marker gene. In this system, DSBs generated by CRISPR/Cas9 at the linker sequence of the tandem repeat will be repaired via single-strand annealing (SSA), a subtype of HDR, resulting in the achievement of antibiotic resistance in plants. This approach allows for a 2- to 23-fold increase in precise and heritable GT efficiency in Arabidopsis and rice. The results indicate that screening with SSA-mediated surrogate system can enrich cells and plants with high HDR activity as well as DSB activity, thus facilitating the establishment of highly efficient GTs at target loci in these plants.

## Introduction

In numerous organisms, homologous recombination (HR)-mediated gene targeting (GT) represents a highly effective approach for precisely modifying genomes [1–3]. This technique, which can generate desired modifications such as sequence knock-ins (KIs) and substitutions, has been utilized to great effect in a variety of contexts [4–6]. However, GT remains a significant challenge, particularly in seed plants, due to the low frequency of HRs and the difficulty of donor template delivery due to the presence of cell wall [7, 8]. The recent advances in the field of engineered sequence-specific nucleases (SSNs) have significantly contributed to gene editing. It has been demonstrated that double-strand breaks (DSBs) induced by these SSNs facilitate GT establishment in organisms [4]. The majority of DSBs generated by these SSNs are repaired by error-prone nonhomologous end joining (NHEJ), resulting in short in-del random mutations at the target site. Only a small proportion of these DSBs are repaired by error-free homology-directed repair (HDR) in the presence of an appropriate donor template (Fig. S1).

In the HDR pathway, DSB ends are resected by the damage-sensing Meiotic recombination 11 (MRE11)-Radiation-sensitive 50 (RAD50)-Nijmegen breakage syndrome 1 (NBS1) complex (MRN complex), in conjunction with C-terminal binding protein interacting protein (CtIP). Subsequently, long-range resections are generated by the 5′ to 3′ exonuclease activities of Exonuclease 1-Bloom helicase (EXO1-BLM). Replication protein A (RPA) coats the single-stranded DNA (ssDNA) overhangs. The replacement of RPA with RAD52 plays a pivotal role in the annealing of homologous sequences within the two DSB ends, thereby facilitating single-strand annealing (SSA) repair. Some reports identify this SSA repair as a subpathway of HDR [9], while others classify it as an alternative to NHEJ [10]. In the event that RPA is displaced by the ATP-dependent DNA recombinase RAD51, the RAD51-coated ssDNA strand will align and pair with homologous DNA, thereby initiating the synthesis of new DNA strands and facilitating the complete repair of the DSB by HDR [9–11]. The RAD51-mediated HDR subpathways are classified into three categories based on the repair donor template and crossover [11]. The first subpathway is ssDNA-templated repair (SSTR), which employs the ssDNA as a repair template and predominantly occurs through noncrossover synthesis-dependent DNA strand annealing (SDSA). Some studies categorize the SSA repair as SSTR [9, 11]. Conversely, dsDNA donor-templated repair (DSTR) is employed when dsDNA is available for DSB repair. This occurs through noncrossover SDSA and the double Holliday junction (dHJ) crossover pathway (Fig. S1). In *Saccharomyces cerevisiae*, Rad52 has been demonstrated to facilitate the assembly of Rad51 filaments onto ssDNA, thereby promoting HR. However, this function is not observed in mammalian RAD52 [11]. The underlying molecular mechanisms of HDR remain largely unknown, particularly in the context of plants [6, 9]. A more profound comprehension of the molecular processes that underpin GT and HDR is required with the utmost urgency. Therefore, further basic research in this area is imperative. While the precise molecular mechanisms remain a topic of ongoing debate, this study was designed and this manuscript was prepared on the assumption that SSA repair can be considered as a subpathway of HDR and as an SSTR [9, 11].

Although SSN-based HDR-mediated GT events have been reported in a wide range of plant species, their efficiency remains low [4, 12]. This extremely low efficiency has hindered the widespread application of GT technology in plants. Therefore, enhancing GT efficiency is a common challenge that requires urgent attention.

A number of methodologies have been the subject of study with the objective of enhancing the efficiency of SSN-based HDR-mediated GT in plants. These methodologies can be classified into four categories, as follows: (i) SSN-mediated DSB frequency, (ii) donor template delivery, (iii) HDR efficiency, and (iv) screening efficiency. (i) With regard to SSN-mediated DSB frequency, the utilization of diverse CRISPR/Cas systems [13–18], the deployment of enhancers for Cas expression [14, 18–20], and the introduction of multiple sgRNAs have been documented to enhance the efficiency of GT [21]. (ii) In donor template delivery, the use of Agrobacterium-derived VirD2 protein fusion Cas9 facilitated the delivery of the donor T-strand to the DSB site, thereby enhancing the efficiency of genome editing in rice [22]. (iii) In HDR efficiency, the efficiency of HDR-mediated GT was enhanced in rice by the inhibition of NHEJ, an antagonistic pathway of HDR [23], and in poplar by the use of Cas9 fused with CtIP and MRE11, pivotal factors in the HDR pathway [24]. (iv) In screening efficiency, two surrogate screening systems have been documented for GT in maize [25] and rice [26]. Both systems are based on the NHEJ mechanism, which is responsible for the activation of the resistance gene. In light of these considerations, we have postulated that an alternative approach could be employed to develop a surrogate screening system for the efficient establishment of GT in plants.

In light of the suboptimal efficiency of HDR-mediated GT in seed plants, we put forth an SSA-based resistance gene activation surrogate screening system that incorporates an sgRNA target site within a direct repeat of a nonfunctional selection marker gene, thereby facilitating efficient screening. A DSB at this target site is then repaired via SSA, which is a subpathway of HDR [9] [11], thereby restoring the activity of the selection marker gene. It is therefore anticipated that this strategy will enrich plants with higher DSB and HDR activity, thereby elevating the relative GT efficiency. The results demonstrated a significant increase in GT frequency, reaching up to 23-fold in *Arabidopsis thaliana* (Arabidopsis) and rice. This SSA-based resistance gene activation surrogate screening system can be used in both Arabidopsis and rice in the same manner as conventional antibiotic (or herbicide) resistance screening marker genes. The fundamental strategy established in the present study can be applied to a wide range of target loci and plant species for precise modification of the host genome sequence.

## Results

### Split Basta resistance gene surrogate screening system for efficient gene targeting through all-in-one strategy

**Figure 1:**
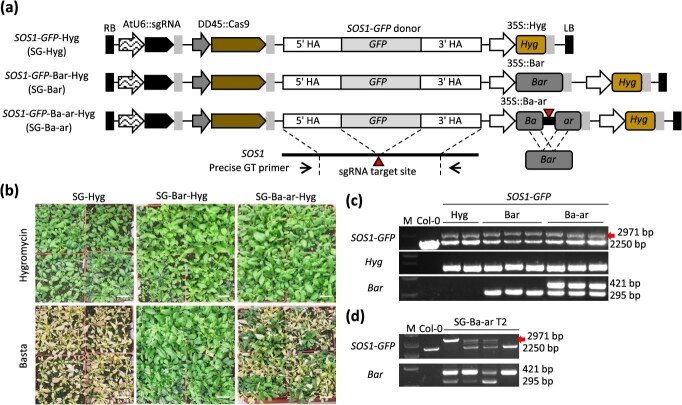
Efficient gene targeting with all-in-one strategy using HDR-mediated Basta resistance gene activation system. (a) Schematic diagram of the *SOS1*-*GFP* all-in-one constructs. (b) Hygromycin- and Basta-resistant phenotypes in *SOS1*-*GFP* all-in-one T1 transformants. T1 seedlings were screened on a 1/2 MS plate containing 50 mg/l hygromycin. The hygromycin-resistant transgenic plants were then transplanted into soil. The scale bars are 3 cm in length. (c) Genotyping in *SOS1*-*GFP* all-in-one T1 transformants. (d) Stable inheritance of *SOS1*-*GFP* GT in the T2 generation. The red arrows indicate the bands corresponding to the *SOS1*-*GFP* GT allele (2971 bp), while the smaller band (2250 bp) signifies the presence of the endogenous *SOS1* allele. The split Ba-ar was detected as a 421-bp band, and the restored intact *Bar* was detected as a 295-bp band. M indicates size marker (c, d).

To investigate the efficiency of GT in Arabidopsis, a series of all-in-one *GFP* KI constructs for an endogenous *Sold Overly Sensitive 1* (*SOS1*) locus were generated (Fig. 1a, Fig. S2a). A substantial body of prior research has demonstrated the *SOS1* locus to be a compelling candidate gene for effective GT establishment in Arabidopsis [27, 28]. Therefore, in this study, we selected SOS1 locus as the target of GT for the model case experiment. The all-in-one construct consists of a DD45 promoter-driven *Streptococcus pyogenes Cas9* (SpCas9; hereafter Cas9), an AtU6 promoter-driven sgRNA cassette, an *SOS1*-*GFP* KI donor flanked by 1-kb homology arms, and antibiotic (and herbicide) resistance selection marker genes (Fig. 1a) [18, 19]. If plants with higher DSB and HDR activity could be enriched using a surrogate selection marker, it is hypothesized that relative GT efficiency would be elevated. To achieve this, a T-DNA construct was designed carrying a split Basta-resistant *Bar* gene with a 100-bp overlapping region in direct orientation (SG-Ba-ar) (Fig. S3). In this system, the functional integrity of the split Ba-ar is compromised due to the presence of a premature stop codon between the Ba and linker sequence. DSB at the Ba-ar linker sequence by Cas9 would be repaired by SSA and functional *Bar* should be restored (Fig. 1a, Fig. S3) [29]. The *SOS1*-*GFP* KI construct with the 35S::Hyg antibiotic resistance gene was used as a control (SG-Hyg). To serve as an additional control, a construct was prepared that harbored both the 35S promoter-driven intact *Bar* and 35S::Hyg genes (SG-Bar) (Fig. 1a).

The three constructs were transformed into the Arabidopsis Col-0 accession, and T1 seedlings were screened on a 1/2 MS plate containing 50 mg/l hygromycin. The hygromycin-resistant transgenic plants were then transplanted into soil (Fig. 1b). More than 400 independent T1 transformants were obtained for each construct in two biological replicates. Genotyping analysis revealed one to three precise and heritable *GFP* KI lines in all constructs (Fig. 1c, Table 1). All hygromycin-positive T1 plants were subjected to Basta screening (Fig. 1b). All 437 and 626 SG-Hyg transformants (including one precise GT-positive line for each) were susceptible to Basta, while the majority (480/484 and 625/629) of SG-Bar lines were resistant (Table 1). Among the 429 and 491 hygromycin-resistant SG-Ba-ar transformants, 200 and 168 of them, including two and one precise GT lines, respectively, survived after Basta selection (Fig. 1b, Table 1). Genotyping by polymerase chain reaction (PCR) and subsequent Sanger sequencing showed that functional *Bar* gene was successfully restored in the Basta-resistant SG-Ba-ar transformants (Fig. 1c, Fig. S2b). These results indicate that CRISPR/Cas9-mediated activation of the Ba-ar system is functional in Arabidopsis and that the Ba-ar system increases relative GT efficiency 2- to 3-fold without false negatives.

**Table 1:** Precise *SOS1*-*GFP* GT efficiency by all-in-one strategy in HDR-based Ba-ar screening system.

Constructs for all-in-one strategy		Hygromycin				Basta			
		Resistant plants	Precise GT	Precise GT efficiency (%)	Fold change (*P*-value)	Resistant plants	Precise GT	Precise GT efficiency (%)	Fold change (*P*-value)
*SOS1*-*GFP*	SG-Hyg	437	1	0.23		0	0		
		626	1	0.16	0	0			
	SG-Bar	484	0	0	1.23 (1.000)	480	0	0	
		629	3	0.48	625	3	0.48		
	SG-Ba-ar	429	2	0.46	1.69 (0.6681)	200	2	1.00	3.33 (0.1688)
		491	1	0.20	168	1	0.60		
	Ba-17-ar	355	1	0.28	1.44 (1.000)	55	1	1.82	7.58 (0.1768)
	Ba-16-ar	411	1	0.24	1.23 (1.000)	6	0	0	
	Ba-14-ar	314	1	0.32	1.64 (0.5403)	1	0	0	

The calculation of GT efficiency was based on the number of individual T1 transformants. Two biological experiments were conducted for SG-Hyg, SG-Bar, and SG-Ba-ar, but not for Ba-17-ar, Ba-16-ar, and Ba-14-ar. The fold change was measured using the average of the biological replicates’ precise GT efficiency. The fold change was calculated for hygromycin-resistant plants (SG-Bar, SG-Ba-ar, Ba-17-ar, Ba-16-ar, and Ba-14-ar) with SG-Hyg as the control, and for SG-Ba-ar and Ba-17-ar with SG-Bar as the control. (See ‘Materials and methods’ for statistical analysis).

**Figure 2:**
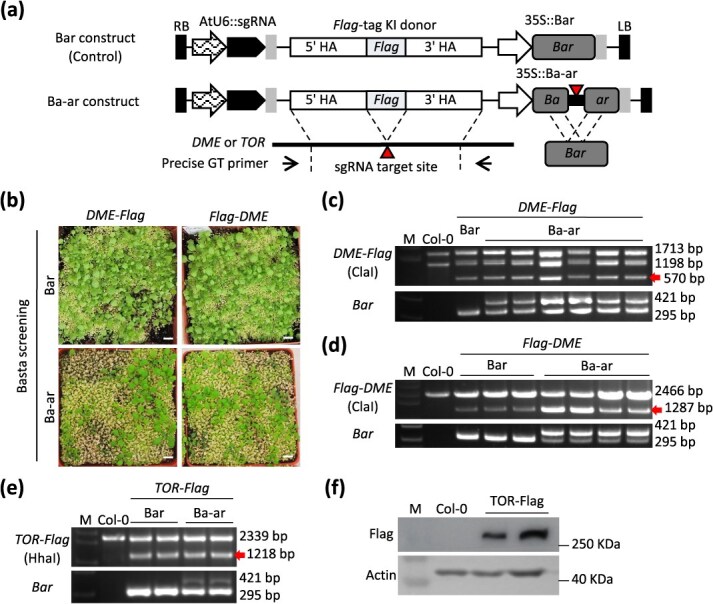
Efficient gene targeting with sequential transformation strategy using HDR-mediated Basta resistance gene activation system. (a) Schematic of *DME*-*Flag*, *Flag*-*DME*, and *TOR*-*Flag* KI constructs for sequential transformation. (b) Basta-resistant phenotype in *DME*-*Flag* (left) and *Flag*-*DME* (right) T1 transformants. The scale bars are 1 cm in length. (c, d) Precise *Flag* epitope tag KI genotyping of the *DME* locus in T1 transformants, *DME*-*Flag* (c) and *Flag*-*DME* (d), respectively. The precise GT events were detected by PCR and followed by restriction enzyme digestion (Fig. S5). The red arrows represent the band corresponding to the precise GT of *DME*-*Flag* (570 bp) (c) and *Flag*-*DME* (1287 bp) (d). (e) Precise *Flag* epitope tag KI genotyping of the *TOR* locus in T1 transformants. The precise GT events were detected by PCR and followed by restriction enzyme digestion (Fig. S6). The red arrow indicates the band representing the precise GT of *TOR*-*Flag* (1218 bp). (f) Detection of TOR-Flag by western blotting. M denotes size markers (c–f).

**Table 2:** Precise GT efficiency with sequential transformation strategies in HDR-based Ba-ar screening systems.

Construct for sequential transformation		Basta-resistant plants	Precise GT	Precise GT efficiency (%)	Fold change (*P*-value)
*DME-Flag*	Bar	288	1	0.35	4.50 (0.0164)
		576	6	1.04	
	Ba-ar	33	1	3.03	
		155	5	3.22	
*Flag-DME*	Bar	384	2	0.52	8.90 (0.0010)
		384	1	0.26	
	Ba-ar	104	4	3.85	
		97	3	3.09	
*TOR-Flag*	Bar	384	2	0.52	23.52 (0.0120)
		288	0	0	
	Ba-ar	18	1	5.56	
		15	1	6.67	
*GBF3-S4A*	Bar	224	13	5.80	5.75 (0.0174)
	Ba-ar	9	3	33.33	
*GBF3-S4D*	Ba-ar	22	2	9.09	
*CPK28-S515D*	Bar	41	1	2.44	4.73 (0.2907)
	Ba-ar	26	3	11.54	
*CPK28-S515A*	Ba-ar	17	3	17.65	

The calculation of GT efficiency was based on the number of individual T1 transformants that were examined. Two biological experiments were conducted for all experimental groups, with the exception of *GBF3*-*S4D* and *CPK28*-*S515A*. The fold change was measured using the average of the biological replicates’ precise GT efficiency with Bar as the control. (See ‘Materials and methods’ for statistical analysis).

All *SOS1*-*GFP* GT events obtained were precise, heterozygous in the T1 generation, and stably inherited by the offspring in accordance with Mendelian inheritance (Fig. 1d), as has been previously reported [14, 18, 19, 27, 30, 31].

### Ba-ar surrogate system for sequential transformation strategy-mediated gene targeting

As previously documented, a sequential transformation strategy, involving the transformation of a donor with an sgRNA construct into a stable Cas9 transgenic parental line, has been shown to improve the GT efficiency of plants [27, 30, 31]. In the present study, the aforementioned parental line, which harbors a DD45 promoter-driven Cas9 (ABRC stock number CS69955), has been employed to transform donor constructs for the sequential transformation strategy-mediated GT (Fig. S4). To explore the broad application of the Ba-ar system, we performed *Flag* epitope tag KI to the C- or N-terminal of the *Demeter* (*DME*) locus (Fig. 2a, Fig. S5). As a control, *Flag*-KI constructs with *Bar* were also prepared. When all T1 seedlings were subjected to Basta screening, the Ba-ar transformants had lower survival rates than the Bar transformants (Fig. 2b). In two biological replicates, at least one precise GT plant was obtained from 288 to 576 *DME*-*Flag* and *Flag*-*DME* control *Bar* T1 transformants, with efficiencies ranging from 0.26% to 1.04% (Fig. 2c, Table 2). On the other hand, precise *Flag*-tag KI plants were obtained when the Ba-ar system was applied, with an efficiency of 3.03%–3.22% for *DME*-*Flag* and 3.09%–3.85% for *Flag*-*DME* (Fig. 2d, Table 2). In comparison to the conventional *Bar* resistance gene, the relative GT efficiency of the Ba-ar system demonstrated a 4- to 8-fold increase for *DME*-*Flag* and *Flag*-*DME*.

*Target Of Rapamycin* (*TOR*) is a long gene, 17 748 bp in genome sequence and 8200 bp in cDNA. Such long size makes molecular genetic experiments difficult. To solve these problems, *Flag*-KI into the C-terminal of the *TOR* gene was attempted using the Ba-ar system (Fig. S6). When the intact *Bar* was applied as a selection marker gene, the efficiencies of the precise GT events ranged from 0% to 0.52%. In contrast, the *TOR*-*Flag* GT showed a >20-fold increase in efficiency when the Ba-ar system was applied (Fig. 2e, Table 2). Not only the precise incorporation of the *Flag* epitope tag sequence was confirmed, but also the detection of the TOR-Flag fusion protein by western blotting (Fig. 2f). These results indicate that the SSA-mediated Ba-ar system is a useful approach to improve the relative efficiency of GT, and that GT is a powerful technique in molecular biology research.

### Ba-ar surrogate screening system for base substitution

Next, we investigated whether the Ba-ar system could improve the efficiency of sequence substitution as well as KI. Two target genes were chosen: *G-Box Binding Factor 3* (*GBF3*) and *Calcium-dependent Protein Kinase 28* (*CPK28*). A total of four donor constructs with 1 kb homologous arms for GT were designed to introduce amino acid substitutions in these two genes (*GBF3*-*S4A*, *GBF3*-*S4D*, *CPK28*-*S515D*, and *CPK28*-*S515A*) (Fig. S7). The *Bar* control constructs yielded precise sequence substitutions of *GBF3*-*S4A* and *CPK28*-*S515D* with efficiencies of 5.8% and 2.44%, respectively (Table 2). Using the Ba-ar system, the efficiency of precise GT events increased by a factor of about five, to 33.33% and 11.54%, respectively (Table 2). In addition, although no control *Bar* constructs were generated, relatively high GT efficiencies of 9.09% and 17.65% were obtained for *GBF3*-*S4D* and *CPK28*-*S515A*, respectively (Table 2).

### HDR-mediated surrogate screening system for NPTII

**Figure 3:**
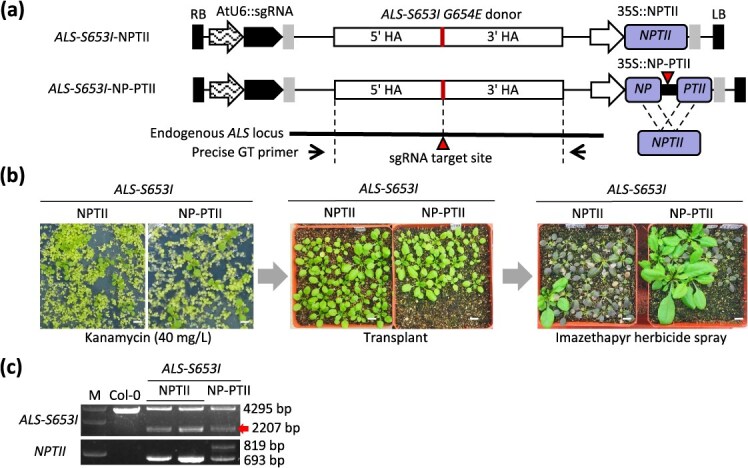
HDR-mediated surrogate screening system for base substitution at the *ALS* locus. (a) Schematic diagram of the *ALS*-*S653I* base substitution constructs. (b) Kanamycin- and imazethapyr herbicide-resistant phenotypes in *ALS*-*S653I* base substitution T1 transformants. The scale bars are 1 cm in length. (c) Genotyping in *ALS*-*S653I* base substitution T1 transformants. The precise GT events were detected by PCR and followed by restriction enzyme digestion (Fig. S9a). The red arrow indicates the precise *ALS*-*S653I* base substitution GT allele (2207 bp), while the larger band (4295 bp) signifies the presence of the endogenous *ALS* allele. The split NP-PTII was detected as an 819-bp band, and the restored intact *NPTII* was detected as a 693-bp band. M denotes size marker.

In addition to the Ba-ar system, we examined the applicability of the same strategy to another antibiotic resistance gene, *neomycin phosphotransferase II* (*NPTII*) (Fig. S8). The *Acetolactate synthase* (*ALS*) gene is an enzyme important in the biosynthesis of branched-chain amino acids in plants [32]. Imidazolinone (IM) herbicides inhibit ALS function, but two amino acid substitutions in ALS (S653I and G654E) confer IM herbicide resistance [33]. ALS amino acid substitution construct for S653I and G654E (*ALS*-*S653I*) were designed in NPTII and NP-PTII backgrounds and transformed into the parental line (Fig. 3a, Fig. S9a). For efficient screening, transformed plants were selected with kanamycin, followed by application of imazethapyr herbicide to select for GT events, which were determined by genotyping (Fig. 3b). Precise base substitution GT plants were obtained in both NPTII and NP-PTII backgrounds (Fig. 3c). A functional *NPTII* gene was restored in kanamycin-resistant NP-PTII plants (Fig. 3c, Fig. S9b). The NP-PTII system successfully increased the efficiency of screening for precise GT events at the *ALS* locus by 2-fold (Table 3). The findings suggest that the SSA-mediated resistant gene activation surrogate screening systems can be universally implemented at numerous selection marker genes to enhance the relative efficiency of GT.

**Table 3:** Precise base substitution GT efficiency of *ALS*-*S653I* in HDR-based NP-PTII screening systems.

Sequential transformation		Kanamycin-resistant plants	IM-resistant plants	Precise GT	Precise GT efficiency (%)	Fold change (*P*-value)
*ALS-S653I*	NPTII	91	9	2	2.20	2.39 (0.1031)
		293	46	5	1.71	
	NP-PTII	28	10	1	3.57	
		52	14	3	5.77	

The calculation of GT efficiency was based on the number of individual T1 transformants that were examined. Two distinct biological experiments were conducted. The fold change was determined by calculating the average of the biological replicates’ precise GT efficiency with NPTII as the control. (See ‘Materials and methods’ for statistical analysis).

### S‌SA-mediated surrogate screening system for sequential transformation strategy-mediated *OsFTL1*-*GFP* GT in rice

**Figure 4:**
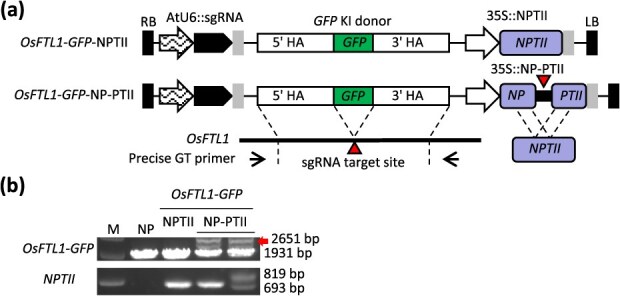
NP-PTII surrogate screening system for *GFP*-KI at the rice *OsFTL1* locus. (a) Schematic diagram of the *OsFTL1*-*GFP* KI constructs. (b) Genotyping in *OsFTL1*-*GFP* KI T1 transformants. The red arrow indicates the precise *OsFTL1*-*GFP* GT allele (2651 bp), while the smaller band (1931 bp) signifies the presence of the endogenous *OsFTL1* allele. The split NP-PTII was detected as an 819-bp band, and the restored intact *NPTII* was detected as a 693-bp band. M denotes size marker.

To demonstrate the versatility of the split-resistance gene activation surrogate screening system, a GT experiment was conducted in rice. Recently, efficient and precise GT by using sequential transformation strategy in rice has been reported [31, 34]. The *FLOWERING LOCUS T-LIKE 1* (*OsFTL1*) gene in rice represents a promising candidate for use in the *GFP*-KI GT approach, as previously documented [31, 34]. Accordingly, the same donor and sgRNA construct utilized in the preceding report was employed for the *OsFTL1*-*GFP* KI control [31, 34]. Furthermore, an examination was conducted to determine whether the NP-PTII system with the *OsFTL1* sgRNA target site would enhance the efficiency of GT (Fig. 4a). The two biological experiments yielded no precise *GFP*-KI GT rice plants when the NPTII control construct was utilized (Table 4). However, two precise and heritable *OsFTL1*-*GFP* GT rice plants were obtained in the second biological repeat experiment for the NP-PTII background, with an efficiency rate of 5% (Fig. 4b, Table 4). It is regrettable that precise heritable *OsFTL1*-*GFP* GT rice plants were not obtained when the NPTII control construct was utilized in the present study. Consequently, the fold change could not be calculated. Nevertheless, the findings indicate that the SSA-mediated resistance gene activation surrogate screening system can be applied to a wide range of plant species.

### Enrichment of plants with high DSB and HDR activity

**Figure 5:**
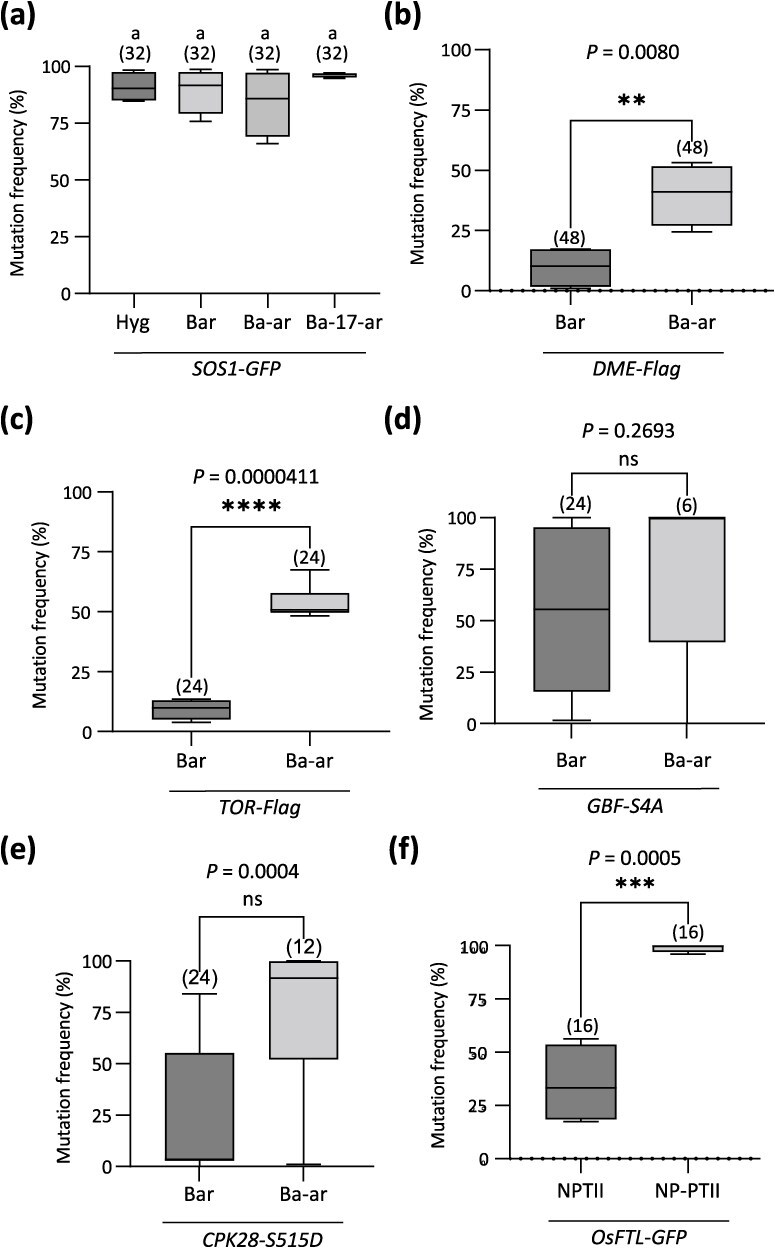
Mutation frequencies at target loci. (a–e) Mutation frequency analysis of *SOS1*-*GFP* (a), *DME*-*Flag* (b), *TOR*-*Flag* (c), *GBF*-*S4A* (d), and *CPK28*-*S515D* (e) target sites in GT-negative T1 Arabidopsis plants. (f) Mutation frequency analysis of *OsFTL1*-*GFP* in GT-negative T0 rice plants. The mutation frequencies in the GT-negative plants were determined in comparison to the Col-0 for Arabidopsis or Nipponbare for rice wild-type control. The number of samples analyzed is indicated in parentheses. The standard deviation of the nonpaired two-tailed Student’s *t*-test was calculated, and the resulting *P*-values were presented.

What plants would be enriched by the SSA-mediated resistant gene activation surrogate screening system is an important question. In order to address this question, an examination was conducted of the mutation frequency of target sites by Cas9 in GT-negative plants [14, 18, 21]. In the case of *DME*-*Flag*, *TOR*-*Flag*, and *CPK28*-*S515D*, the mutation frequency was markedly elevated by the Ba-ar system (Fig. 5b, c, e). Similarly, in rice, the mutation frequency at the *OsFTL1*-*GFP* target site was markedly elevated in NP-PTII plants relative to the control NPTII plants (Fig. 5f). The results demonstrate that plants exhibiting elevated DSB activity were enriched through the utilization of SSA-mediated surrogate screening systems for resistant gene activation. In contrast, there was no change in the mutation frequencies at the *SOS1* locus among the Hyg, Bar, and Ba-ar lines before and after Basta screening (Fig. 5a). Similarly, a significant enrichment of mutation frequency was not observed in Ba-ar in comparison with Bar for *GBF*-*S4A* samples (Fig. 5d). This is thought to be due to the mutation rate approaching a plateau. The results may indicate that screening with SSA-mediated activation systems can enrich cells and plants with high HDR activity as well as DSB activity, thus facilitating the establishment of highly efficient GTs at target loci in these plants.

### Further improvement by truncation of sgRNA target site

The present study demonstrated that the SSA-mediated surrogate resistance gene activation systems enhanced the relative GT efficiency at all target sites examined. However, the improvement in GT efficiency of *SOS1*-*GFP* by the all-in-one strategy was lower than expected. Following the screening of Basta in the SG-Ba-ar transgenic plants, approximately half of the T1 transgenic plants survived (Table 1). Furthermore, the enhancement of precise GT for the *ALS*-*S653I* substitution in the NP-PTII background was also less pronounced (2- to 3-fold) than at other loci (Table 3). This observation may be attributed to the DSB activity of the designed sgRNA, rather than to the difference between the all-in-one or sequential transformation strategies, or between the Ba-ar or NP-PTII selection marker gene itself. Mutation frequency at the *SOS1* locus almost reached a plateau, with no further improvement made by the Ba-ar system (Fig. 5a). Therefore, although a slight improvement, this improvement can be attributed to enriched HDR activities.

As described above, the activation of the split-resistance gene surrogate screening system is primarily contingent upon the activity of sgRNAs and SSA, which is considered a subpathway of HDR and an SSTR [9, 11], efficiency. To achieve efficient establishment of GT, it is essential to ensure a high level of DSB activity of the sgRNA [27]. However, if the DSB activity of the sgRNA is markedly elevated, the efficiency of activation of the split-resistance gene will also be increased, resulting in a reduction in the relative GT efficiency. To address this discrepancy, truncated sgRNA target sites were employed at the linker sequence of the Ba-ar system for the *SOS1*-*GFP* all-in-one strategy (Fig. S10). As a consequence of the reduction in the DSB activity of CRISPR/Cas9 resulting from the shortened sgRNA recognition site [35], it is hypothesized that the relative GT efficiency will be increased by the use of a truncated sgRNA target site. Following hygromycin screening of a series of truncated target site constructs, a single precise *GFP*-KI GT strain was obtained for each construct, exhibiting an efficiency of 0.24%–0.32%. The vast majority of plants with 16- and 14-bp truncated target linker sequence (Ba-16-ar and Ba-14-ar), including those with precise GT positive, were eliminated by Basta screening (Table 1). The results indicate that the activity of DSB by CRISPR/Cas9 was decreased due to the shortened target linker sequence [35], and that the restoration of functional *Bar* was unsuccessful in these plants. In contrast, the efficiency of precise GT increased 7.5-fold in 17-bp target site plants (Ba-17-ar) following Basta screening, with no instances of false negatives (Table 1). The mutation ratio of the *SOS1* target site in Ba-17-ar GT-negative plants is comparable to that observed in other Bar and Ba-ar systems (Fig. 5a). It can thus be concluded that the employment of the truncated target site at the linker sequence, which results in a reduction in the DSB efficiency of the split-resistance gene, allows for the further improvement of the relative GT efficiency in plants.

## Discussion

The present study describes the development of an SSA-mediated resistance gene activation surrogate screening system for the efficient establishment of GT in plants. The SSA-based resistance gene activation surrogate screening system comprises an sgRNA target site within a direct repeat of a nonfunctional selection marker gene, facilitating efficient screening. A DSB at this target site is then repaired via SSA, which is a subpathway of HDR [9]. This process restores the activity of the selection marker gene. The screening system demonstrated variable improvements in the efficacy of GT. The magnitude of the enhancements varied from 2- to 23-fold among target sites. However, it is important to note that not all of these enhancements were statistically significant. The results of this study indicate that this strategy can enrich plants with not only higher DSB activity, but also higher HDR activity, thereby elevating the relative GT efficiency. This suggests that GT technology will be employed on a regular basis in the future for use in plants. Furthermore, all GT events obtained in the present study were precise, exhibiting no mutations in the homology arms and KI sequences, and were stably inherited by the offspring in accordance with Mendelian inheritance, as previously reported [14, 18, 19, 27, 30, 31].

Surrogate systems based on the restoration of defective resistance marker genes are currently being developed to efficiently enrich mutant plants through the CRISPR/Cas or base editing systems [26, 36–38]. In Arabidopsis, the simultaneous mutagenesis of the *GLABRA2* (*GL2*) gene, which is associated with the trichome phenotype, with Cas9 resulted in an increased frequency of mutant lines in the T1 generation [39]. Similarly, the generation of mutations by Cas9 at the *Multi-Antibiotic Resistance 1* (*MAR1*) locus and subsequent kanamycin screening enhanced the efficiency of mutagenesis in tomato and Arabidopsis [40]. The simultaneous base substitution at the *ALS* locus using a base editor also has the potential to enhance the efficiency of the screening process in wheat [38].

**Table 4:** Precise GT efficiency of rice *OsFTL1*-*GFP* in HDR-based NP-PTII screening systems.

Sequential transformation		Kanamycin-resistant plants	Precise GT	Precise GT efficiency (%)	Fold change (*P*-value)
*OsFTL1-GFP*	NPTII	57	0	0	
		36	0	0	(0.2066)
	NP-PTII	63	0	0	
		40	2	5	

The calculation of GT efficiency was based on the number of individual T1 transformants that were examined. Two biological experiments were performed. However, the fold change could not be measured because precise GT efficiencies were zero for the NPTII control. (See ‘Materials and methods’ for statistical analysis).

Moreover, it has been documented that the surrogate screening systems have been employed not only for mutagenesis, but also for the establishment of GT in plants. In the case of maize, an activation system for the herbicide-resistant *Hra* gene via Cas9-mediated excision of the donor sequence for efficient GT screening has been reported [25]. In a parallel development, a surrogate screening system for KI via NHEJ in rice has been engineered, wherein Cas9-mediated mutagenesis was strategically employed to restore resistance to hygromycin by rescuing a nonfunctional *HPT* gene [26]. Alternatively, the trichome phenotype resulting from the *GLABRA1* (*GL1*) gene has been utilized for the assessment of GT events in Arabidopsis [41]. In addition, it has been reported that simultaneous mutagenesis of the above-mentioned endogenous *MAR1* gene can achieve up to a 4-fold increase in the efficiency of GT in Arabidopsis [28]. While these visible phenotypes and antibiotic resistance are useful for efficient GT event screening, it should be noted that all of these reports rely on NHEJ repair for efficient screening. However, there is a lack of documented evidence regarding the enrichment of plants with higher HDR activity. The hypothesis put forth is that a system based on HDR can facilitate a more efficient establishment of GT in plants. In light of the aforementioned rationale, this study has developed an SSA, which is a subpathway of HDR-mediated [9] activation resistance gene surrogate screening system. In the course of preparing this manuscript, a comparable surrogate screening system via SSA for enriching DSB-susceptible plants in rice and Brassica was reported for efficient mutagenesis [42]. The present study demonstrated that the SSA-mediated activation resistance gene surrogate screening system can enrich plants with higher HDR activities as well as higher DSB activities. Consequently, we hypothesize that this could be the primary reason that precise and heritable GT plants can be obtained with great efficiency.

In previous reports, we have put forth the hypothesis that single-stranded T-DNA (ssT-DNA, also known as T-strand) released from Agrobacterium would be the most probable candidate molecule for the template of GT in plants [14, 18, 27, 31]. Furthermore, the delivery of ssT-DNA by means of VirD2 fusion Cas9 has been demonstrated to enhance GT efficiency in rice [22], thereby providing additional support for this hypothesis. These findings suggest that the Agrobacterium method-mediated GT could be established via SSTR, which employs the ssDNA as a repair template and predominantly occurs through noncrossover SDSA in plants. The initiation of both the SSA and SDSA pathways is contingent upon the recognition of the DSB site and resection by the MRN complex and CtIP (Fig. S1). This is followed by long-range end resection, which involves EXO1 and BLM. The RAD51 assembly of long 3′ ssDNA overhangs initiates homologous recombination-based DNA repair, including SADA. Conversely, RAD52 has been demonstrated to facilitate SSA in mammalian cells [10, 11, 43]. The majority of factors involved in the HDR pathway are highly conserved in the plant kingdom [44]. It is notable that two distinct RAD52 orthologue genes are present within the plant kingdom [45]. The overexpression of AtRad52-1A was demonstrated to enhance SADA, while concurrently suppressing SSA in Arabidopsis [46]. The existence of a distinctive mechanism within the HDR pathway in plants may be suggested; however, the HDR pathway itself appears to be highly convergent among organisms. In light of the aforementioned considerations, it seems likely that the split-resistant genes are activated via the SSA pathway, thereby establishing precise GT via SDSA in the context of this study. As both the SSA and SDSA repair pathways are initiated by the same mechanism (Fig. S1), it is possible to enrich the plants with higher DSB and HDR activities, thereby improving the relative GT efficiency observed in the present study. The molecular mechanisms underlying the process by which cells and plants with higher HDR activity are selected by using SSA-mediated resistant gene activation surrogate systems are not yet fully understood. This is primarily attributable to the absence of molecular evidence, with the exception of mutation frequency analysis. The complexity of analyzing individual egg cells and early embryonic cells that have undergone HDR-mediated GT in the parent plants is a primary factor contributing to this phenomenon. This is further compounded by the remarkably low efficiency of GT establishment. However, further research is necessary to elucidate the underlying molecular mechanisms of HDR-mediated GT establishment in plants.

GT has proven to be a powerful and versatile tool for both fundamental research and molecular breeding applications. For example, *OsFTL1*-*GFP* KI rice plants have been instrumental in elucidating the molecular function and spatiotemporal dynamics of OsFTL1, a key regulator of inflorescence development. OsFTL1 facilitates the transition from vegetative to inflorescence meristem and modulates panicle architecture by enhancing determinacy in distal meristems [34]. In a similar manner, *CPK28*-substituted Arabidopsis lines are being utilized to elucidate the mechanisms underlying plant aluminum resistance, while *TOR*-*Flag* KI plants will serve as a valuable resource for the functional characterization of TOR signaling. In addition, recent findings have demonstrated that the tolerance of abiotic stresses in plants can be enhanced through the precise integration of stress-responsive *cis*-regulatory elements into the promoter regions of select genes [47].

According to the findings of preceding studies, GT-mediated modifications have been shown to generally not disrupt endogenous gene expression or epigenetic regulation, such as DNA methylation patterns [19, 27, 30, 31]. Consequently, the absence of detectable epitope-tagged proteins in certain GT lines may be indicative of low endogenous expression levels of the target gene. However, there is documented evidence of instances where KI sequences have been shown to interfere with gene expression or protein function. For instance, *YFP* KIs in *AFL1* and *PSC1*, as well as *bar* KI in *RPS5A* in Arabidopsis, have been observed to exhibit such interference [18, 48]. These observations underscore that, while GT is not without limitations, it remains an indispensable and highly effective research tool. Moreover, it is anticipated that GT will be utilized in molecular breeding to enhance plant traits in the near future.

Overall, our SSA-mediated resistance gene activation surrogate system offers a potential strategy for improving the screening efficiency of precise and heritable GT events in plants, with observed enhancements reaching up to 23-fold under certain conditions. The fundamental principle of this system can be widely expanded to other marker genes, including *GFP* and *HPT*, as well as to other plant species [42]. In light of these findings, it can be posited that the SSA-mediated resistance gene activation system may prove particularly efficacious in establishing precise and heritable GTs in plants with high relative efficiency.

## Material and methods

### Gene accession numbers

*SOS1*, At2g01980; *DME*, At5g04560; *TOR*, At1g50030; *GBF3*, At2g46270; *CPK28*, At5g66210; *ALS*, At3g48560; *DD45*, At2g21740; *OsFTL1*, LOC_Os01g11940.

### Plant materials and growth condition

*Arabidopsis thaliana* (Arabidopsis) parental line DD45-#58 (Arabidopsis Biological Resource Center (ABRC) stock number CS69955) was used in the sequential transformation strategy mediated gene targeting (GT) by *Agrobacterium tumefaciens* (GV3101), and Col-0 (Columbia-0) accession materials were also employed. Plants were grown under long-day conditions (16 hours of light and 8 hours of darkness) at 22°C, with one-month-old plants used for transformation and seed collection conducted when plants were two months old. A soil mix composed of peat moss, vermiculite, and perlite in a 2:1:1 proportion, with a pH of approximately 6.5, was used for cultivation. Balanced liquid fertilizer was applied every two weeks, and sterile conditions were implemented during the transformation. Seeds were sterilized using sodium hypochlorite and grown on agar plates before being transplanted into the soil.

### Plasmid construction

The sgRNA was designed and driven by the AtU6–26 Pol III promoter, based on sgRNA design websites including CRISPR Primer Designer (http://plantsignal.cn/CRISPR/crispr_primer_designer.html), CRISPR-PLANT (https://www.genome.arizona.edu/crispr/index.html), CRISPOR (http://crispor.tefor.net/), CHOPCHOP (http://chopchop.cbu.uib.no/), DESKGEN (https://www.deskgen.com/landing/cloud.html), and CRISPR tool ATUM (https://www.atum.bio/eCommerce/cas9/input). The generation of GT constructs for the all-in-one and sequential transformation strategy was in accordance with the publications [30, 49]. In brief, an AtU6–26 promoter-driven sgRNA cassette and donor sequence were constructed in pCambia1300 with Bar or Ba-ar resistance gene. The human codon-optimized *Streptococcus pyogenes* Cas9 was utilized for the parental line and the all-in-one construct [30, 49]. The constructed vector was then transferred into *A. tumefaciens* GV3101 for subsequent transformation experiments in planta. The complete list of primers utilized in this study can be found in the Table S1.

### Plant transformation

Arabidopsis was transformed via the Floral Dip method using *A. tumefaciens*-carrying destination vectors. Transformed T1 generation seeds were screened using the Basta method, in which seeds were spread on soil and 0.02% Glufosinate Ammonium solution (SANGON) was applied three to four times. For hygromycin-based screening, T1 seeds were grown on a 1/2 MS plate containing 50 mg/l hygromycin. Surviving seedlings were transferred to the soil for further cultivation and genotyping. One week later, Basta sprays were applied three to four times at 3-day intervals using a 0.2% Basta solution. For the *ALS*-*S653I* base substitution GT screening, T1 seeds were germinated on a 1/2 MS plate containing 50 mg/l kanamycin. Surviving seedlings were transferred to soil, and 2 mg/l imazethapyr was applied via spray, as previously reported [14].

The japonica rice cultivar Nipponbare (*Oryza sativa*) was utilized in the present study. The rice plants were cultivated at 28°C in soil with a 12-h light/12-h dark photoperiod in a greenhouse. The Agrobacterium-mediated transformation of rice was conducted in accordance with the methodology previously reported [31, 50]. The selection of transformants was conducted by applying 25 mg/l G418.

### DNA analysis

To extract total genomic DNA, leaf tissues were ground to a fine powder in liquid nitrogen using the ShakeMaster AUTO (Bio Medical Science Inc., Tokyo, Japan). Then, DNA was extracted from the grounded leaf tissue by the cethyltrimethyl ammonium bromide (CTAB) method for individual plant analysis. The extracted DNA was subsequently utilized for the PCR analysis of GT events. The primers used for genotyping were designed using the Primer3 (https://primer3.ut.ee/) (Table S1). The PCR system utilized 2× Taq Plus Master Mix II (Vazyme, Nanjing, China), in accordance with the manufacturer’s instructions. The PCR products were then separated by electrophoresis on a 1.5% (w/v) agarose gel and were subjected to visualization using Image Lab software (Bio-Rad Laboratories, Hercules, CA, USA). The Sanger sequencing method was then employed to determine the engineered genomic sequence.

Precise GT events were identified through the use of PCR. The design of full-length primer sets was intended to facilitate annealing upstream and downstream of the homology arms, thereby ensuring the amplification of both endogenous and KI alleles (Fig. 1a, d, Figs S1 and S3a, b). Consequently, as previously reported, the detection of GT events by full-length primer sets indicates that these GTs are at least heterozygous, precise, and stably inherited to progenies [27, 31].

The TIDE website (https://tide.nki.nl) was employed to ascertain the mutation frequency of the target sites [50]. Subsequently, the PCR amplicons derived from these target sites underwent Sanger sequencing. The mutation frequencies observed in the GT-negative plants were then compared to the Col-0 for Arabidopsis or Nipponbare for rice wild-type control. The standard deviation of the nonpaired two-tailed Student’s *t*-test was calculated, and the resulting *P*-values are presented. To ascertain the mutation frequency, the intact *Bar* and *NPTII* samples were utilized as a control.

In instances where groups involved biological experiment replicates, a one-way analysis of variance was conducted within each group prior to intergroup testing. To ascertain the statistical significance of the precise GT events, the two-tailed Fisher’s exact test was utilized. The hypothesis of differences between the various experimental groups was evaluated through the aforementioned method.

### Western blotting

The total proteins of leaves were dissolved in a 2× SDS loading buffer. The protein samples were separated using a 10% SDS-PAGE gel and transferred onto PVDF membranes. The membranes were then blocked with 5% skim milk for 2 h and incubated overnight at 4°C with primary antibodies of HRP-mouse anti-Actin antibody (Abclonal, 1:1000 dilution) or anti-Flag antibody (Abclonal, 1:1000 dilution). The membranes were then washed three times with TBST. Protein signals were detected with Clarity Western ECL Substrate (Bio-Rad) using the ChemiDocXRS imaging system (Bio-Rad).

### Statistical analysis

The selection of statistical tests was based on the sample size (*n*) and the expected frequencies (T). Pearson’s chi-square test was employed when *N* ≥ 40 and all expected counts satisfied T ≥ 5. For datasets with *N* ≥ 40 where at least one expected frequency fell in the range 1 ≤ T < 5, Yates’ continuity-corrected chi-square test was applied. Fisher’s exact test was used in three scenarios: Firstly, when *N* ≥ 40 and two or more expected frequencies were between 1 ≤ T < 5. Secondly, when the total sample size was small (*N* < 40). Thirdly, when any expected count was below T < 1. Furthermore, in instances where the chi-square test yielded a *P*-value marginally close to 0.05, Fisher’s exact test was adopted to ascertain the robustness of the findings.

## Supplementary Material

Web_Material_uhaf196

## Data Availability

The plasmids harboring the Ba-ar and NP-PTII have been deposited with Addgene and are available from their website (Addgene IDs 226 815 and 226 816, respectively). All data supporting the results of this study are presented in the manuscript, including Supplementary information. The datasets generated or analyzed during the current study are available from the corresponding author on reasonable request.
