# Supplementary material for: SSA-mediated selection marker gene activation enhances relative gene targeting efficiency in plants
Source: Hortic Res. 2025 Nov 5;12(11):uhaf196. doi: 10.1093/hr/uhaf196 (PMC12596130; doi:10.1093/hr/uhaf196)
Supplement: Web_Material_uhaf196 [file web_material_uhaf196.zip › Supporting Information-ba-ar-v4.pdf]

Supporting Information

Supplementary Figure legends

Supplementary Figure S1. DNA double-strand break repair pathways mediated by NHEJ and HDR.

The schematic diagram illustrates the simplified DSB repair pathways via NHEJ and HDR. The 3' overhang single-stranded DNA (ssDNA) plays a crucial role in the process of HDR. The direct repeat coated with RAD52 is repaired by single-strand annealing (SSA), which represents a subpathway of HDR. The RAD51-mediated HDR subpathways are classified into three categories based on the nature of the repair donor template and the mechanism of crossover. These categories are ssDNA-templated repair (SSTR), dsDNA donor-templated repair (DSTR) via non-crossover synthesis-dependent DNA strand annealing (SDSA), and DSTR via the double Holliday junction (dHJ) crossover pathway.

Supplementary Figure S2. *SOS1-GFP* GT construct, target locus, and primers.

(**a**) Schematic diagram of the *SOS1-GFP* KI GT donor construct and details of the endogenous *SOS1* locus. The *GFP* KI fragment of *SOS1-GFP* was flanked by homology arms of 1 Kbp each. Arrows indicate full-length primers used to detect GT events. The protospacer-adjacent motif (PAM) is indicated in red. (**b**) Sequence results of the Ba-ar system. The upper panel shows Sanger sequencing result of *SOS1-GFP* Ba-ar system construct. The lower panels represent Sanger sequencing results of the functionally restored *Bar* gene in *SOS1-GFP* Ba-ar T1 transgenic plants.

Supplementary Figure S3. Working principle of Ba-ar system.

The schematic diagram shows the detailed working principle of the Ba-ar system. An sgRNA generates DSBs at both the endogenous target locus and at linker sequences in the Ba-ar system. The sgRNA target sequences were cloned into a multicloning site located downstream of the premature stop codon of the nonfunctional Ba-ar linker of each construct. The DSB generated in the Ba-ar linker sequence by Cas9 should be repaired by SSA, which is a subpathway of HDR, restoring a functional *Bar*.

Supplementary Figure S4. Flowchart of gene targeting by sequential transformation strategy.

Schematic representation of sequential transformation strategy-mediated gene targeting in plants. The parental line is characterized by the presence of DD45::Cas9 or ZmUbq1::Cas9 transgenes, which have been integrated into the genomes of either Arabidopsis or rice, respectively. The donor construct, which carries a donor sequence with homology arms, an sgRNA targeting the endogenous locus of interest, and an SSA-mediated surrogate screening marker gene, is then transformed into the parental line. The obtained transformants were then subjected to screening by the selection marker, a process consistent with standard transformation procedures, either on plates or in soil. The precise GT events were determined by PCR-based screening.

Supplementary Figure S5. *Flag* KI GT constructs, *DME* target locus and primers.

(**a**, **b**) Schematic representation of *DME-Flag* (**a**) and *Flag-DME* (**b**) GT donor constructs and the endogenous *DME* locus. The donor constructs carried a KI Flag sequence flanked by two homology arms of 800 bp each. Arrows indicate the full-length primers used to detect GT events. PAM sequences are shown in red letters; ATG and stop codons are shown in blue letters. **C**, Genotyping by PCR and restriction enzyme digestion of *DME-Flag* and *Flag-DME* using full-length primer sets. The location of the ClaI restriction enzyme sites and fragment size after digestion are shown.

Supplementary Figure S6. *TOR-Flag* GT construct, target locus and primers.

(**a**) Schematic representation of *TOR-Flag* GT donor construct and the endogenous *TOR* locus. The *Flag* KI fragment of *TOR-Flag* was flanked by homology arms of 1 Kbp each. Arrows indicate the full-length primers used to detect precise GT events. PAM sequence is shown in red letters; stop codon is shown in blue letters. (**b**) Genotyping by PCR and restriction enzyme digestion of *TOR-Flag* using full-length primer sets. The location of the HhaI restriction enzyme site and fragment size after digestion are shown.

Supplementary Figure S7. Base substitution GT constructs and target *GBF3* and *CPK28* loci.

(**a**, **b**) Endogenous target *GBF3* (**a**) and *CPK29* (**b**) loci and nucleotide substitution GT donor constructs sequences. Blue squares indicate the position of the intended amino

acid base substitution. PAM sequences are shown in red letters and nucleotide substitutions are shown in light green letters.

Supplementary Figure S8. HDR-mediated activation of NP-PTII selection marker.
The schematic diagram shows the detailed working principle of the NP-PTII system. The sgRNA target sequences were cloned into a multicloning site located downstream of the premature stop codon of the nonfunctional NP-PTII linker. The DSB generated in the NP-PTII linker sequence by Cas9 should be repaired by SSA, restoring a functional *NPTII*.

Supplementary Figure S9. *ALS-S653I* base substitution GT construct, target locus and primers.
(**a**) Schematic representation of *ALS-S653I* base substitution GT donor construct and the endogenous *ALS* locus. The *ALS-S653I* base substitution was flanked by homology arms of 2 Kbp each. Arrows indicate the full-length primers used to detect precise GT events. Blue square indicate the position of the intended amino acid base substitution. PAM sequences are shown in red letters and nucleotide substitutions are shown in light green letters. PvuI restriction enzyme, indicated by orange square, was used to detect the precise GT events. (**b**) Sequence results of the NP-PTII system. The upper panel shows Sanger sequencing result of *ALS-S653I* NP-PTII system construct. The lower panels represent Sanger sequencing results of the functionally restored *NPTII* gene in *ALS-S653I* NP-PTII T1 transgenic plants.

Supplementary Figure S10. Truncation of sgRNA target sequence at the Ba-ar linker.
The provided schematic diagram illustrates the shortened sgRNA target site within the Ba-ar linker for use with the *SOS1-GFP* KI construct. The various lengths of truncated sgRNA target sequences were cloned into a multicloning site located downstream of the premature stop codon of the nonfunctional Ba-ar linker. The PAM sequences are indicated by red text.

Supplementary Figure S11. Original uncropped gel pictures.

Supplementary Table S1. Sequences of primers used in this study

Primer name	Primer sequence (5' – 3')
**sgRNA**	
SOS1-GFP	GTTAGAAGGTGATAATGCGGcgg
DME-Flag	CAACAAAACCTAAAGATGACtgg
Flag-DME	GAATTCGAGGGCTGATCCGGggg
TOR-Flag	GATGACCCATCTCACCAGAAagg
GBF3-S4A/D	TCCTATGGGAAATAGCAGCGagg
SPK28-S515D/A	GAAGATTCCTGTGACCTGCAggg
ALS-S653I	GTGTTGCCGATGATCCCGAGtgg
OsFTL1-GFP	CAGAGGGAGTCTGGAACCGGagg
**Cloning promoters**	
DD45 promoter	AAATGTTCCTCGCTGACGTAAGAAGAC
	AAATGTTCCTCGCTGACGTAAGAAGAC
**GFP donor construction**	
GFP	ATGGTGAGCAAGGGCGAGGAG
	TTACTTGTACAGCTCGTCCATGCCGT
Flag	ACAAGTTTGTACAAAAAAGCAGGC
	CCCAGCTTTCTTGTACAAAGTGTG
**Genotyping**	
SOS1-GFP Precise GT	CCTCAGGTTGAGAGTCTTGTC
	AAGATAACAATTGATCGGTTGAGTG
DME-Flag Precise GT	GTACAGCTAGAGCTAAAAAGCATTT
	CGCGTGTTGGATAAGTTCGA
Flag-DME Precise GT	GGTGCAATTAGAAACGAACAT
	CACTCCCGGTTTCTTTAGAT
TOR-Flag Precise GT	CCCTAAGACGATGCCAACTC
	GGTTGGTTATATTCTTGGGTTAGGT
GBF3-S4A/D Precise GT	GTCGACAGAAGTGCTCTCCG
	GATCGTTTATGTTCATCTGCACC
SPK28-S515D/A Precise GT	GCTAACTGCTGCACAAGCAC
	TGTCTGCCATCGTCGATATCAG

ALS-S653I Precise GT	TTCTCAGCCACAAATTCTACATTT
	TTAATGTCTGAGAAAGGCGAAAG
OsFTL-GFP Precise GT	GACAATAATCCTTGAACCACCTTC
	CAATCGTAATCGTAGAGTGTAGGC
Bar	TGCACCATCGTCAACCACTAC
	TCAGCAGGTGGGTGTAGAGC
Hyg	GCACTGACGGTGTCGTCCATC
	GTCTCCGACCTGATGCAGCTC
NPITT	TTGGGTGGAGAGGCTATTCG
	CGATACCGTAAAGCACGAGG

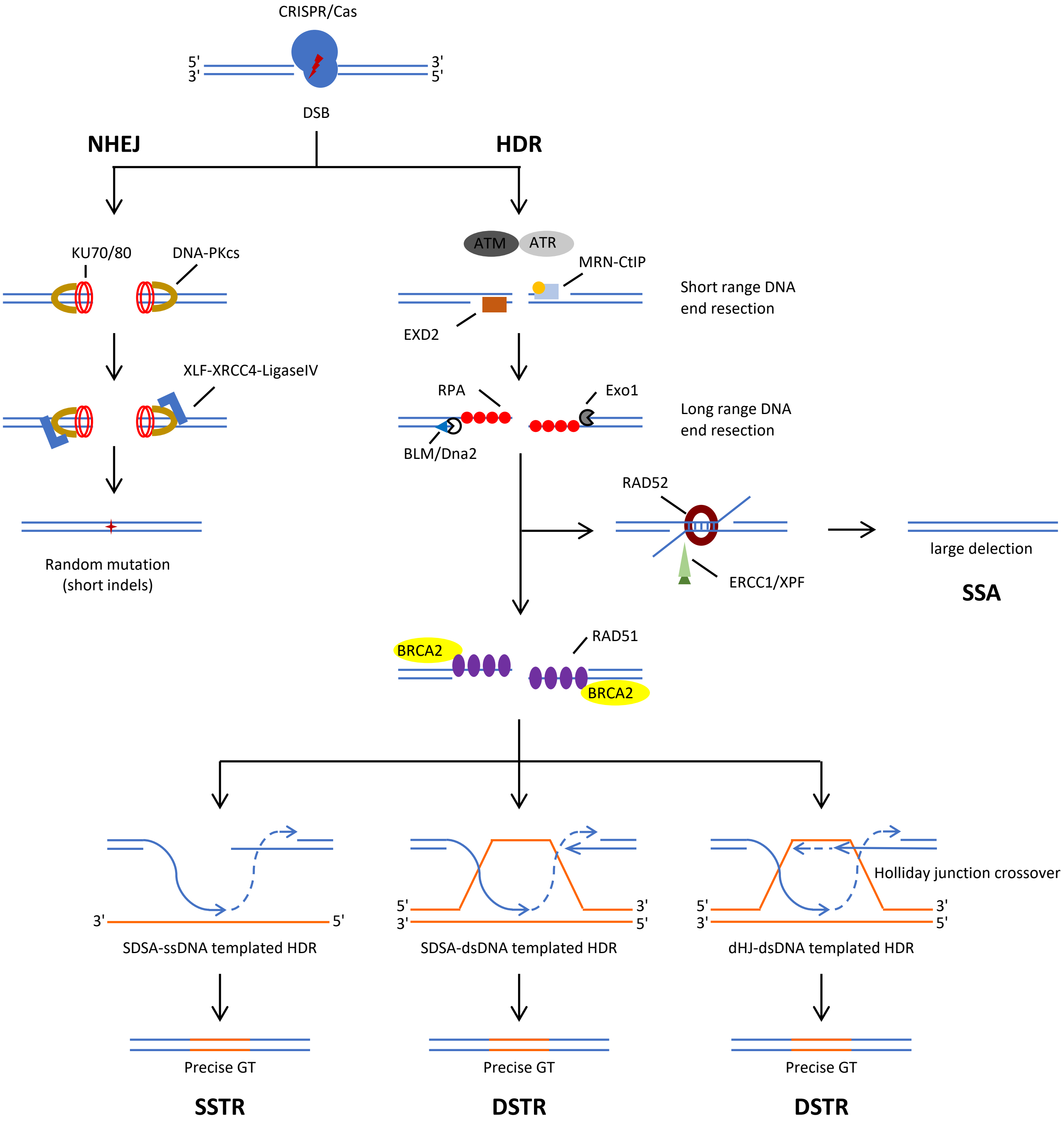

Supplementary Figure S1. DNA double-strand break repair pathways mediated by NHEJ and HDR.

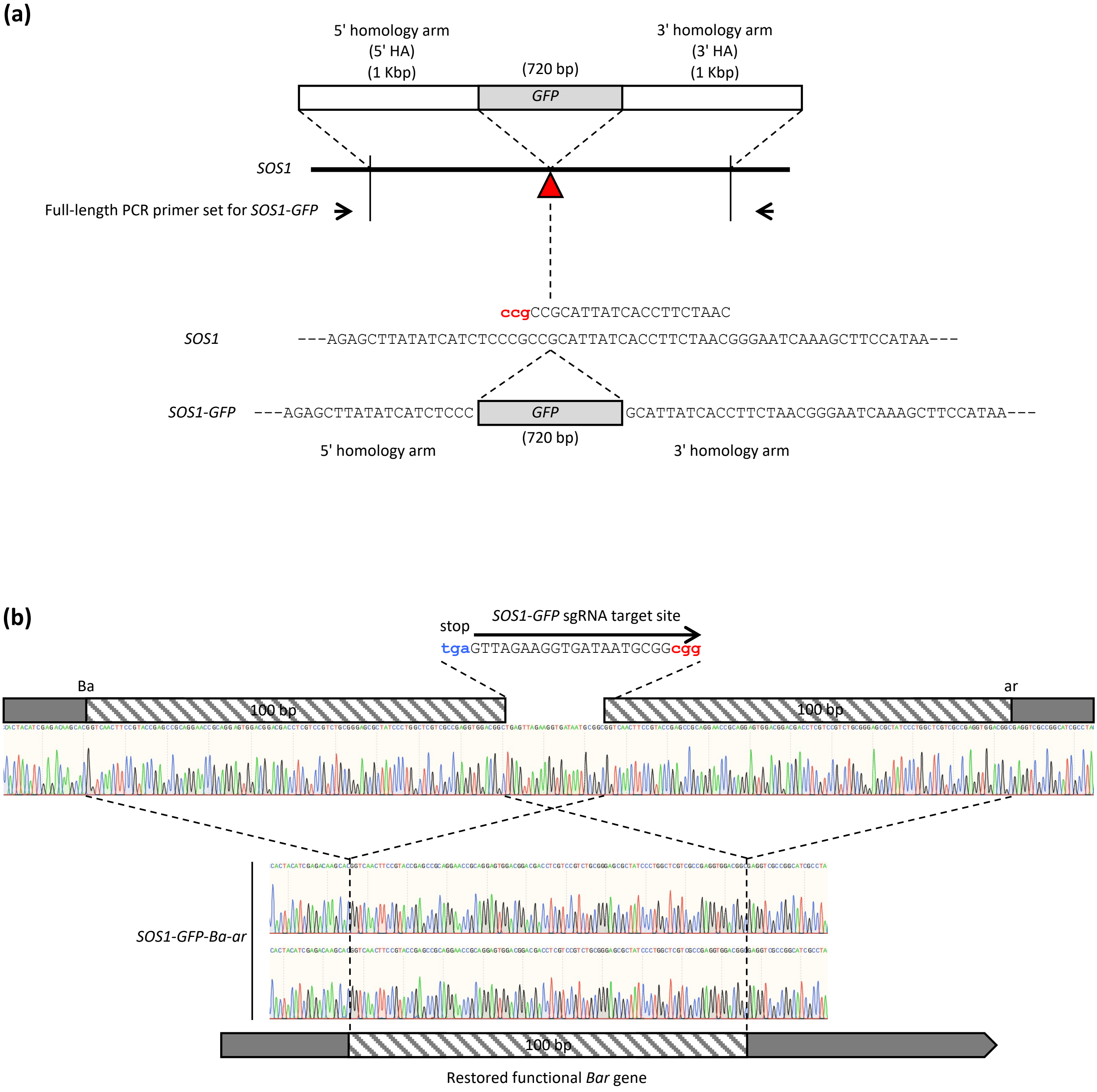

Supplementary Figure S2. ***SOS1-GFP*** **GT construct, target locus and primers.**

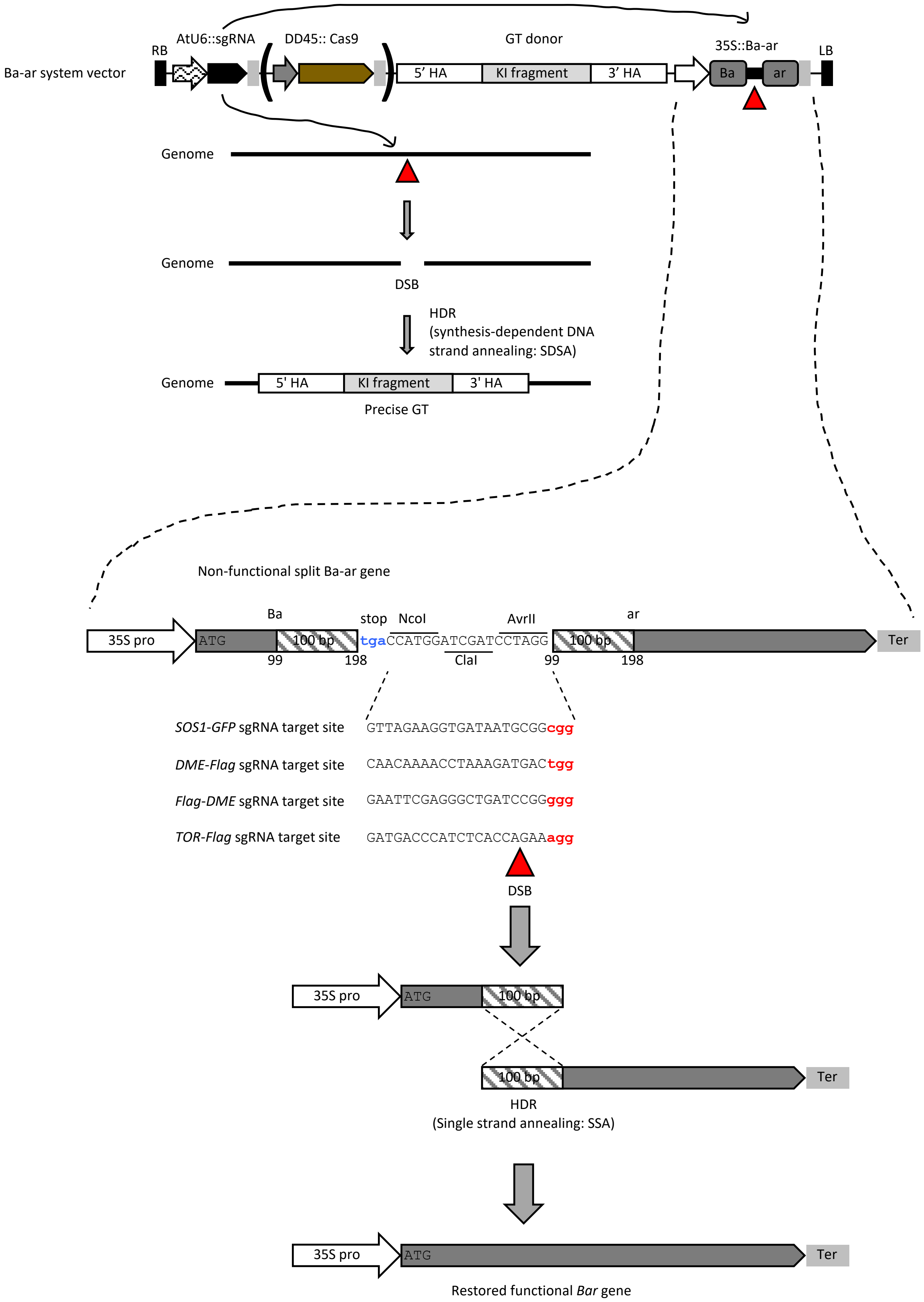

Supplementary Figure S3. Working principle of Ba-ar system.

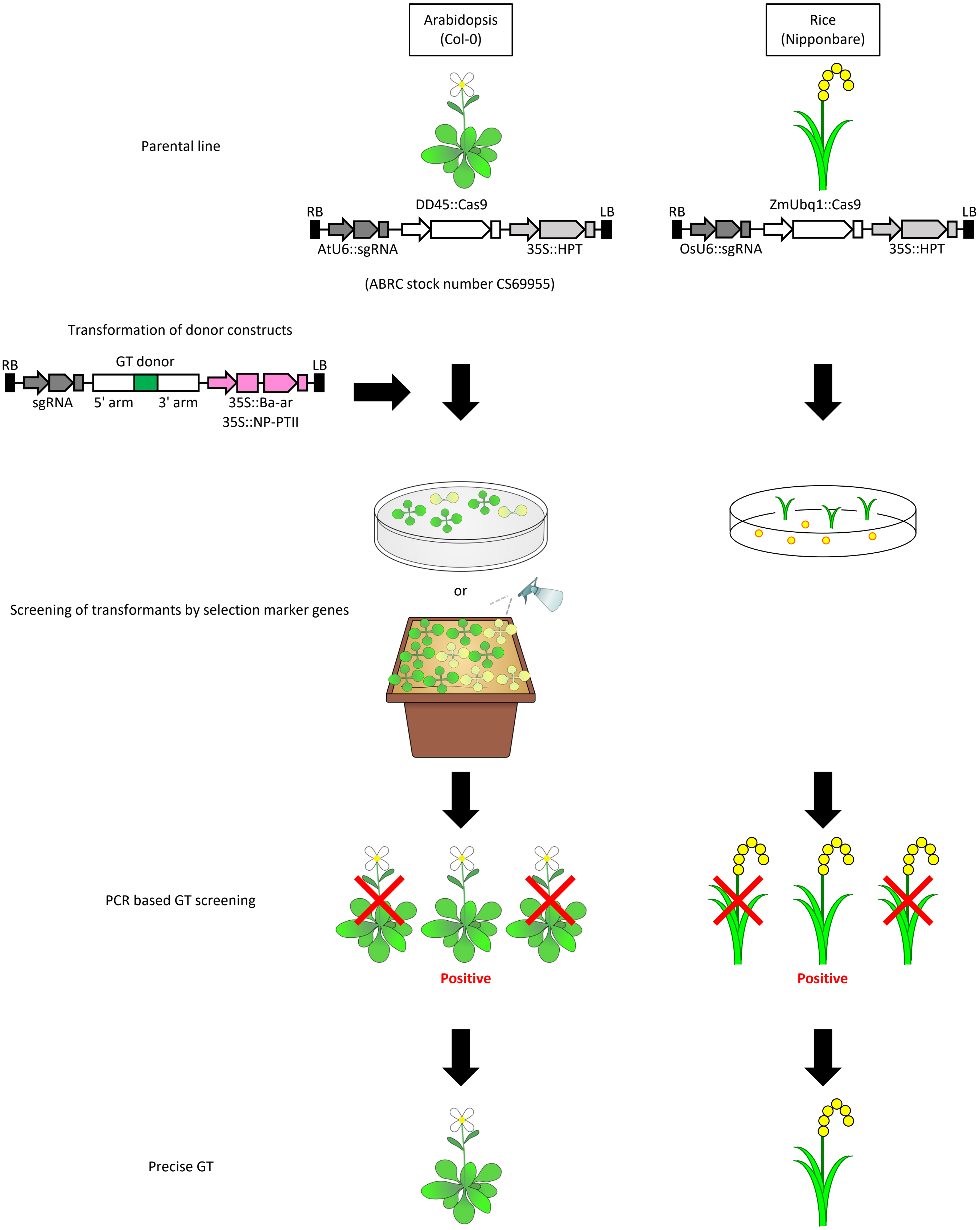

Supplementary Figure S4. Flowchart of gene targeting by sequential transformation strategy.

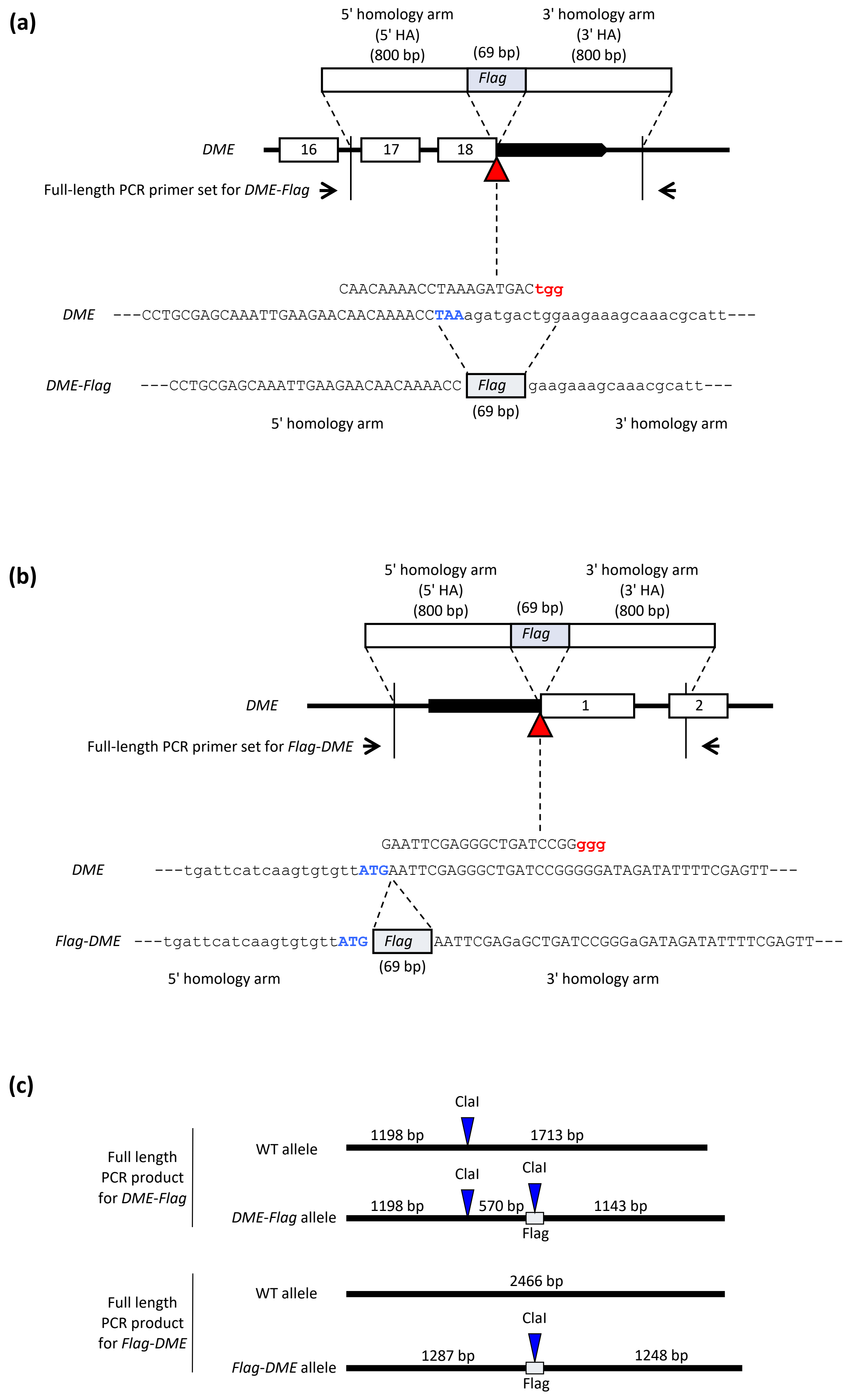

Supplementary Figure S5. ***Flag*** **KI GT constructs,** ***DME*** **target locus and primers.**

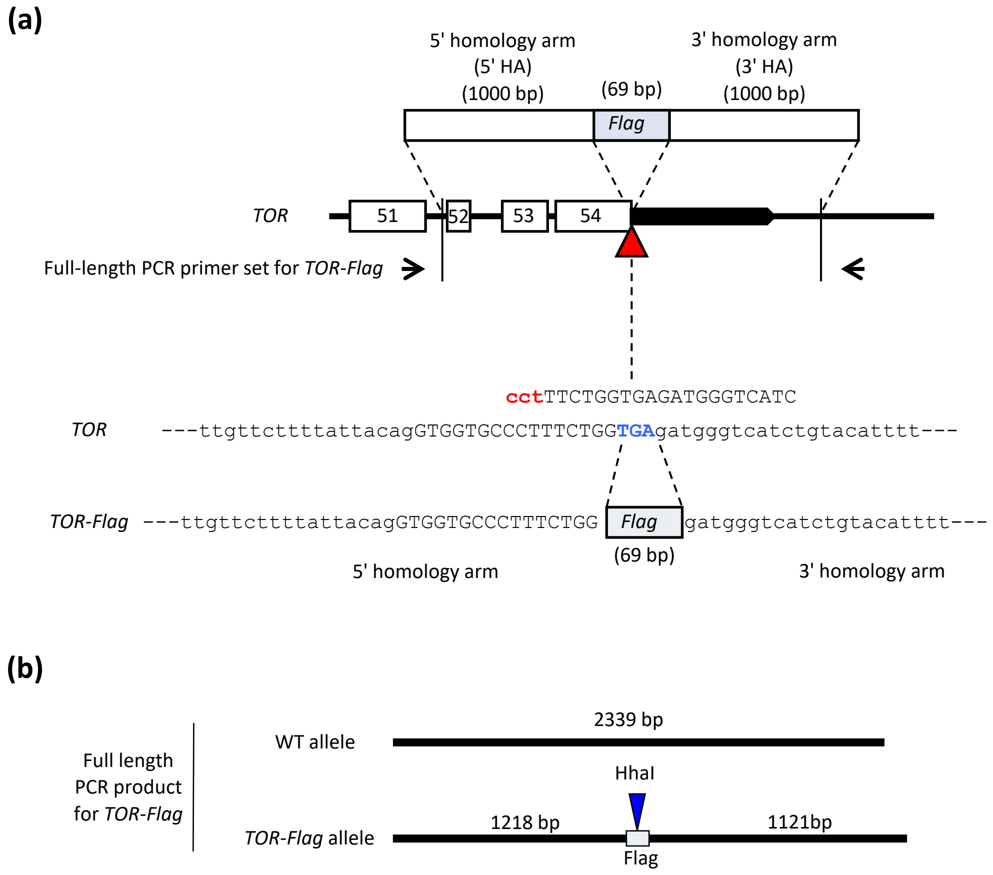

Supplementary Figure S6. ***TOR-Flag*** **GT construct, target locus and primers .**

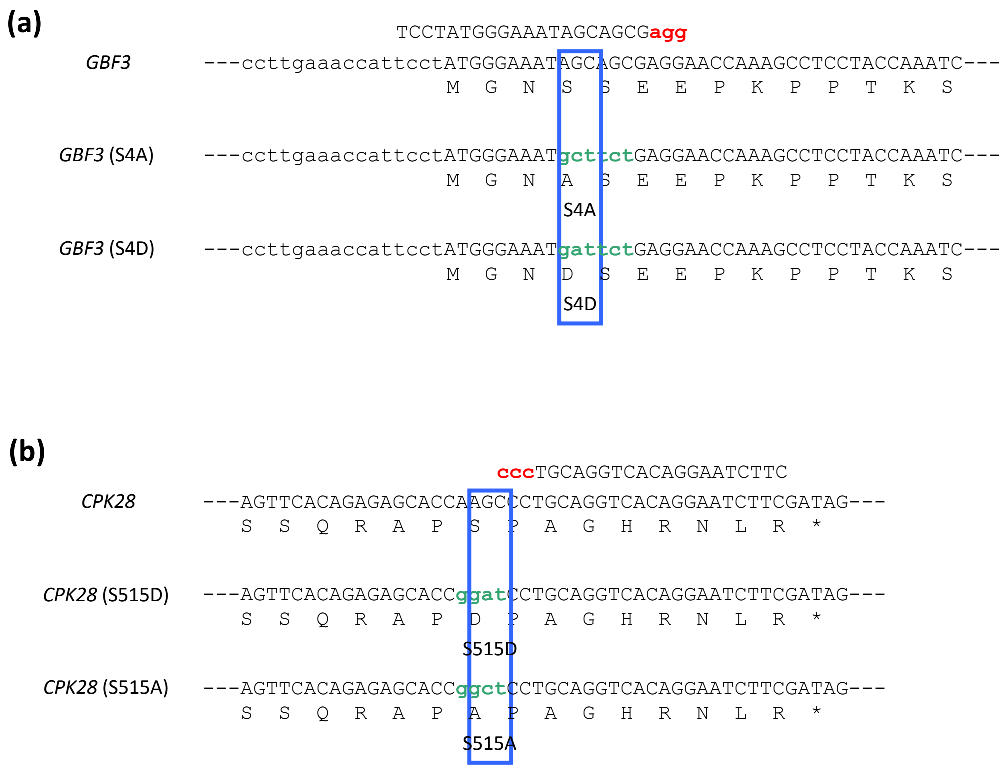

Supplementary Figure S7. Base substitution GT constructs and target *GBF3* and *CPK28* loci.

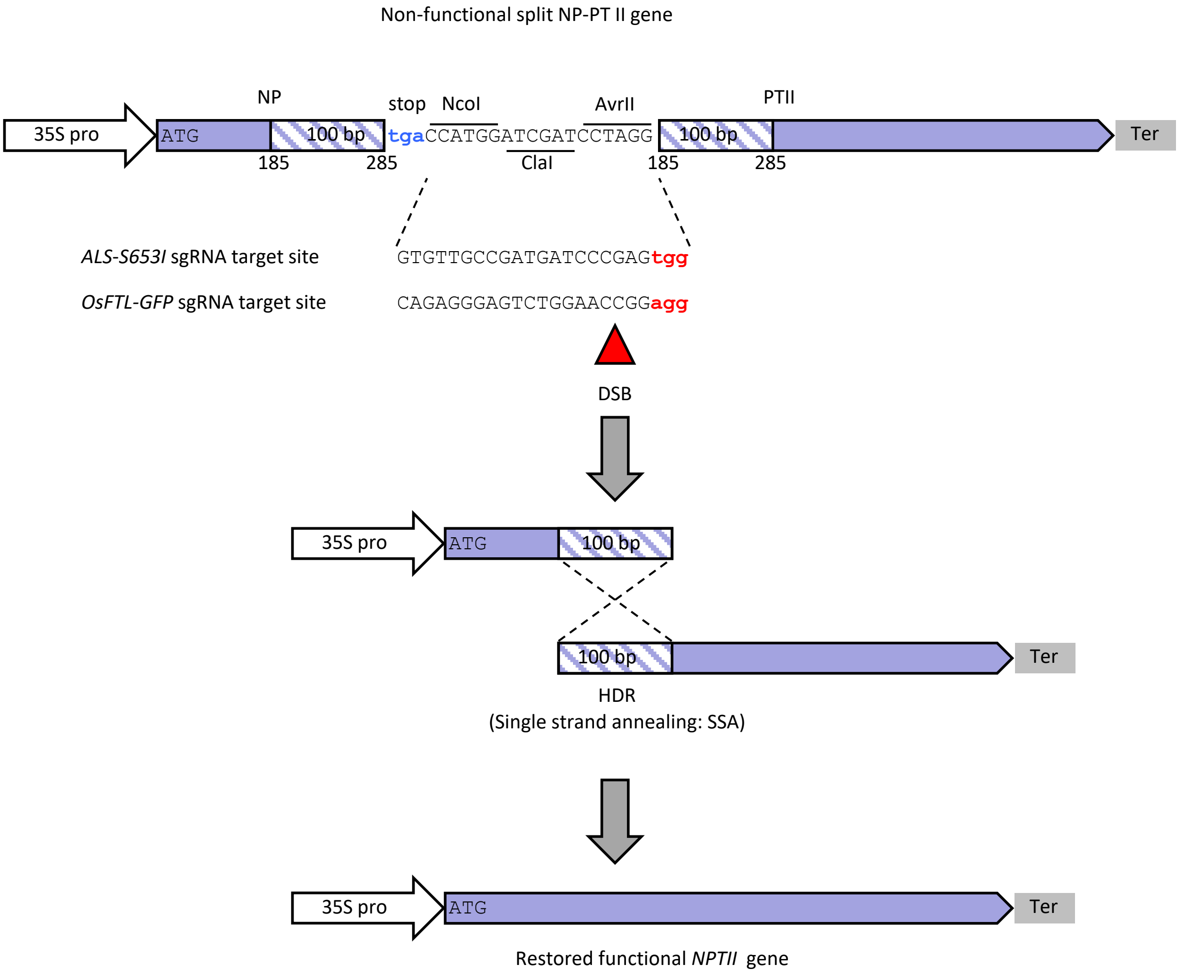

Supplementary Figure S8. HDR-mediated activation of NP-PTII selection marker.

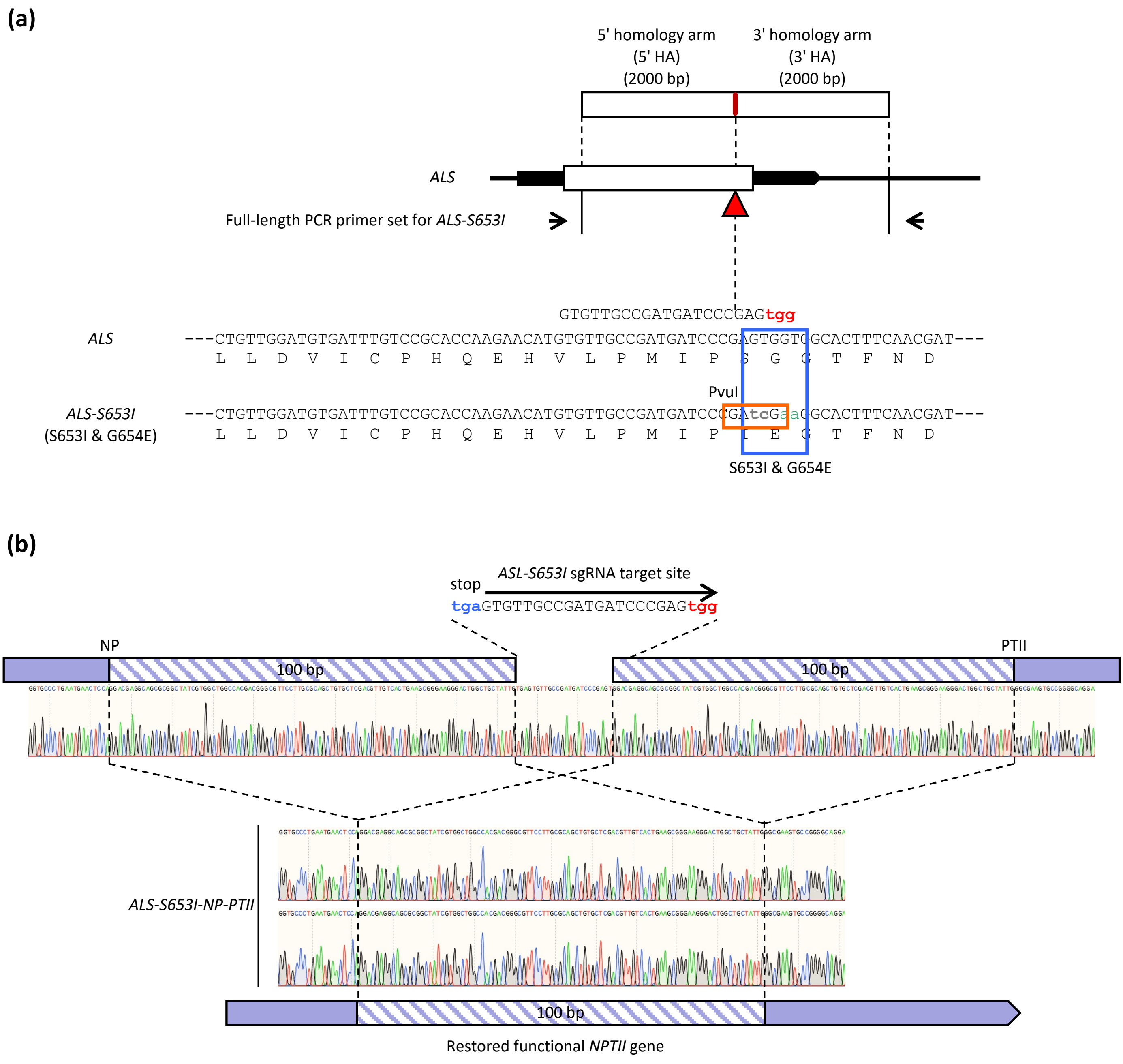

Supplementary Figure S9. ***ALS-S653I*** **base substitution GT construct, target locus and primers.**

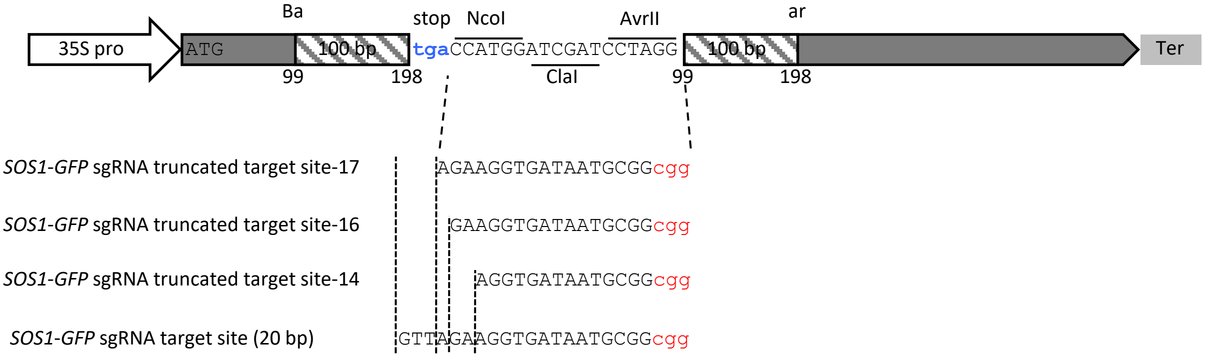

Supplementary Figure S10. Truncation of sgRNA target sequence at the Ba-ar linker.

Figure 1c

SOS1-GFP
(Full-length primer set)

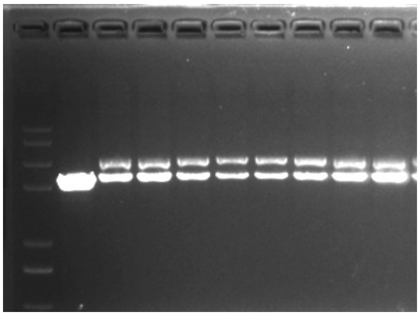

SOS1-GFP
Hyg

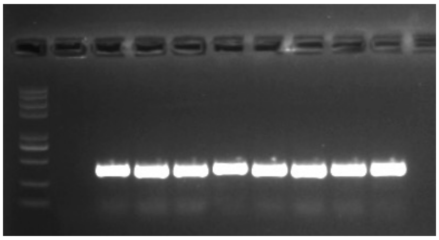

SOS1-GFP
Bar

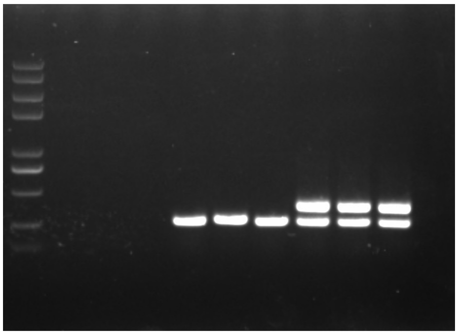

Figure 1d

SOS1-GFP
(Full-length primer set)

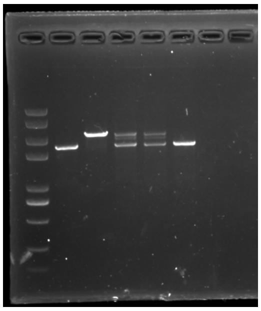

SOS1-GFP
Bar

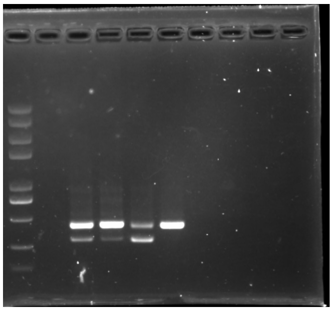

Supplementary Figure S11. Original uncropped gel pictures.

Figure 2c

DME-Flag
(ClaI)

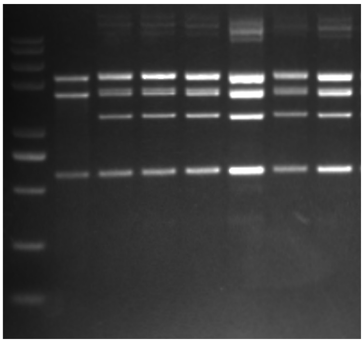

DME-Flag
Bar

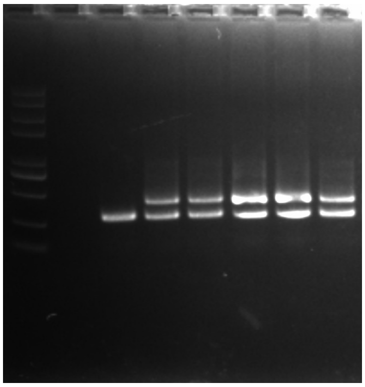

Figure 2d

Flag-DME
(ClaI)

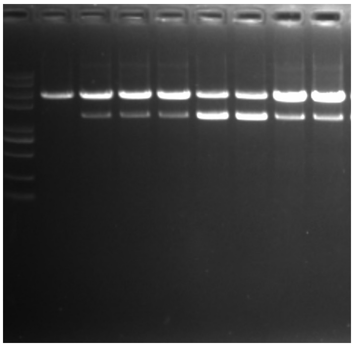

Flag-DME
Bar

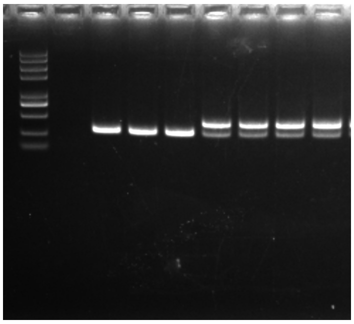

Figure 2e

TOR-Flag
(HhaI)

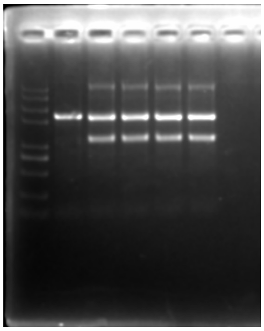

TOR-Flag
Bar

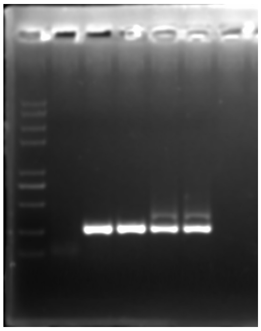

Figure 2f

TOR-Flag

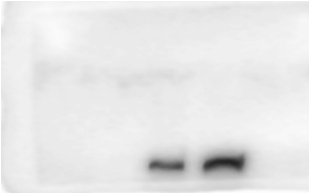

Actin

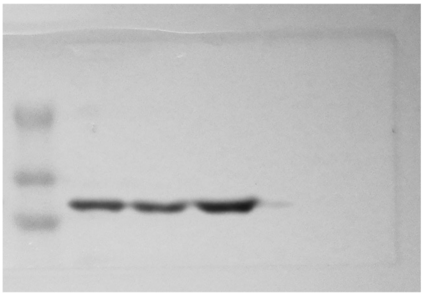

Supplementary Figure S11. Original uncropped gel pictures.

Figure 3c

ALS

NPTII

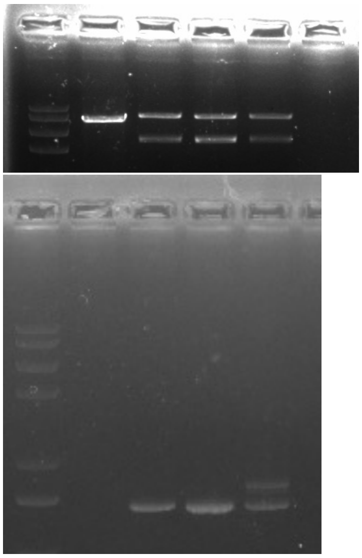

Figure 4b

OsFTL-GFP
(Full-length primer set)

OsFTL-GFP
NPTII

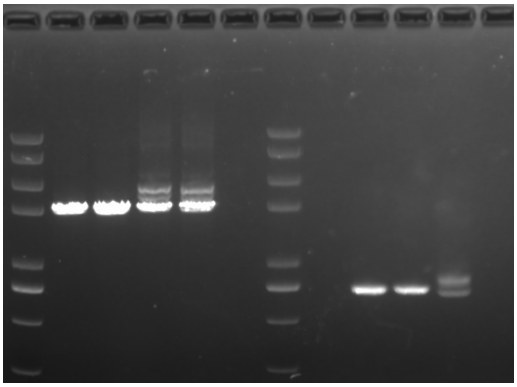

Supplementary Figure S11. Original uncropped gel pictures.
